# The Spread of Disease by Civilisation

**Published:** 1906-11-24

**Authors:** 


### The Spread of Disease by Civilisation.

That government must be conducted in the interests of the governed, is an axiom familiar and grateful to Anglo-Saxon ears. Whatever others may say on the matter, the British peoples believe that the dominance which, as a nation, they exercise over so many subject races is directed by the principle which this axiom defines. In some recent remarks made by Sir Lauder Brunton at the University of London a new application of this principle is emphasised, and the application is one which depends for its success upon medical research and practice. It is true that the pioneer of civilisation, and the Pax Britannica which has followed his advent, have brought to many countries relief from numerous practices which involved a horrible sacrifice of human life. Wars and murders and slavery have thus been made to cease. But the advent of the white man and the establishment of settled government have not been an unmixed boon. "We bring them measles, tracts, and gin," sang a satirical versifier, and it is to the suggestion contained in the first of these three terms that Sir Lauder Brunton now directs attention. Taking the progress of sleeping sickness as an illustration of his definition of the extent of national responsibility, he shows how this disease was formerly limited to the Western part of Africa, and how by the establishment of trade routes and other methods of communication it has now been widely spread over the African continent. Merely as a question for the national conscience it is impossible to escape responsibility for this terrible result of the progress of civilisation.

When these facts are presented to the British people in their true colours and relationships, it can hardly be doubted that the nation will insist on the prosecution of all measures necessary to stay the plague. The difficulty is to secure such adequate presentation. The outbreak of some "sort of war" between a native tribe and the advancing white man, cases of interference with tribal rights, the execution of rebels, or specific examples of cruelty and injustice—these and their like offer themselves as opportunities to politicians for an attack on the Government of the day. In this way they are able to secure national attention, and a chance is obtained for the national conscience to assert itself. But the steady and continuous cruelty and massacre resulting from the progress of the sleeping sickness and other diseases, serve no end in the struggle for party supremacy, and offer no chance for parliamentary advertisement. Hence the horror of the position is hardly heard of in the country generally, and no demand arises for a vigorous attempt to combat it. None the less the facts proclaim a duty to humanity and place on the shoulders of those familiar with them a responsibility which must not be evaded. That responsibility falls, in the first place, on the medical profession, and more particularly on those who can speak with special authority and whose words have ready access to the guides and leaders of public opinion. It cannot, perhaps, be anticipated with confidence that representations of this order will prove successful. There are, unfortunately, examples not a few which point to an opposite conclusion. All the same, there is a plain call of duty in this matter, and we venture to emphasise its demand and to suggest persistence and perseverance in its presentation. Even those who are deaf to a general claim and who feel but a languid sympathy for African natives perishing under the attack of mysterious and fatal diseases, may be aroused by the announcement that the sleeping sickness now threatens to spread to Egypt and Cairo. Such an announcement involves possibilities that even the most careless cannot contemplate with equanimity. Nor is it any longer possible for the European to comfort himself with the assurance that sleeping sickness affects only natives of Africa. The exact contrary is known. Only a few months have passed since a courageous and brilliant young English officer who was engaged in investigating this terrible disease, fell a victim to its deadly onset.

The responsibilities arising from the spread of tropical disease along the track of advancing civilisation demand, however, something more than recognition. It is necessary also that an effort should be made to discharge them. But inquiries of this order mean the expenditure of money, and it is the main purpose of this article to insist that the provision of this money is a duty which the nation cannot avoid. The claims of humanity and the danger of neglect alike urge the necessity of making a determined attempt to establish control over diseases which have been spread by the movements of civilisation, and which, further, carry a risk to civilisation itself.

